# Barriers to epilepsy care in Central Uganda, a qualitative interview and focus group study involving PLWE and their caregivers

**DOI:** 10.1186/s12883-019-1398-z

**Published:** 2019-07-17

**Authors:** Mark Kaddumukasa, Haddy Nalubwama, Martin N. Kaddumukasa, Samden Lhatoo, Nelson Sewankambo, Elly Katabira, Martha Sajatovic, Carol Blixen

**Affiliations:** 1 Department of Medicine, School of Medicine, College of Health Sciences, Makerere University, P.O. Box 7072, Kampala, Uganda; 2 School of Public Health, Makerere University College of Health Sciences, P.O. Box 7072, Kampala, Uganda; 3 McGovern medical School and Health Science Center at Houston, The University of Texas, Houston, USA; 4 Neurological and Behavioral Outcomes Center, University Hospitals Cleveland Medical Center & Case Western Reserve University School of Medicine, 11100 Euclid Avenue, Cleveland, OH 44106 USA

**Keywords:** Epilepsy, Seizures, Barriers, Sub –Saharan Africa

## Abstract

**Background:**

Epilepsy is a common neurological disease with substantial impact on the subject and their caretakers. This exploratory study identified barriers to care for persons living with epilepsy (PLWE) to develop a culturally acceptable nurse led self-management intervention for PLWE previously developed in the United States.

**Methods:**

The study involving 48 participants (31 PLWE and 17 caregivers) with in depth interviews and focus groups was conducted. We obtained insights into barriers to care in PLWE and their caregivers. Using a thematic analytic procedure emphasizing the dominant themes the qualitative responses were analyzed. Median age of PLWE was 24 years (IQR 19–30), and10 (52.6%) were male. The median age of epilepsy onset was 12 years (IQR 6–18), range of 1–37 years. The median age of caregivers was 50 years (IQR 45–50.5), with a range of 18–78 years. Seventy five percent of caregivers (6/8) were females.

**Results:**

Three major areas of perceived barriers involving individual, family or community and provider and healthcare system barriers to epilepsy care emerged. Individual factors like limited epilepsy knowledge and medication non-adherence were reported to be key barriers to epilepsy care. Caregiver burden and lack of family support as well as poor health care access were identified from the family and health care systems.

**Conclusions:**

The main barrier to epilepsy care is limited epilepsy knowledge in PLWE and their caregivers. Improving epilepsy care awareness and knowledge within communities and appropriate health care provider service for epilepsy would help reduce epilepsy barriers and improve care.

## Background

Epilepsy is a brain disorder that affects people of all ages. Worldwide, over 70 million people are estimated to be living with epilepsy, with approximately 80% residing in developing countries [1, 2]. Limited knowledge on epilepsy in PLWE and their caregivers to guide appropriate choices of care remains a challenge, despite advancements in treatment, diagnosis and care of PLWE in our settings. This subsequently portends to recurrent seizures and associated stigmatizing or negative beliefs, and attitudes among community dwellers to PLWE [3–6].

Whereas, variations exist in attitudes, perceptions within countries and cultural social context exist; development of collective strategies and interventions to improve quality of life in PLWE is urgently needed [7, 8]. In other similar studies in Uganda, majority of the respondents had negative beliefs such as attributing epilepsy to supernatural spirits, heredity, and preference of traditional medicine and healers to conventional medicine as a treatment for epilepsy [9–11]. Studies assessing the barriers to epilepsy care in Uganda are not available yet developing a culturally acceptable and effective intervention requires an understanding of these modifiable barriers in epilepsy care. This study therefore set out to explore the perceived barriers to epilepsy care in PLWE and their caregivers in a Ugandan setting.

## Methods

### Study setting and design

The study was conducted at Mukono Municipality, health center IV, epilepsy clinic within greater Kampala, Uganda. The clinic serves as a district clinic for Mukono and the surrounding districts.

This was a cross-sectional qualitative study with a total of 48 study participants were recruited in this qualitative study. For qualitative research, this sample size is within the recommended number of 20–50 individuals [12, 13].

Inclusion criteria for participation in this study was, age 18 years or older, cognitively intact, and able to provide informed consent. We excluded all subjects who were unable to provide written informed consent and those with secondary epilepsy due to traumatic brain injury or stroke.

### Focus group methods

Six [6] separate Focus Group Discussions (FGDs) were conducted consisting of four groups with PLWE (*n* = 19) and two with caregivers (*n* = 8). An experienced qualitative researcher fluent in both English and Luganda (local language of the study area) moderated the FGDs. The FGDs were conducted to give in-depth insights into the perceived barriers of epilepsy care. The in-depth interviews’ were conducted at the respondents’ homes while the FGDs were conducted at the local village chairperson’s residence. We conducted in-depth interviews in 12 PLWE previously discharged from Mulago national referral hospital in Kampala, and nine caregivers of PLWE.

The median age of PLWE was 24 years (IQR 19–30), with a range of 18–41 years, 10 (52.6%) were male. The median age of epilepsy onset was 12 years (IQR 6–18), range of 1–37 years. Only one of the female PLWE was married, with 95% (18/19) unmarried. Two of the PLWE were students while 26% (5/19) were unemployed. The majority of PLWE (12/19) attained a secondary level of education.

The median age for caregivers was 50 years (IQR 45–50.5), with a range of 18–78 years. Seventy five percent of caregivers (6/8) were female. The majority of caregivers had some source of income or held a dedicated form of employment; only two (2/8) were unemployed.

### Qualitative data collection and analysis

Using both face-to-face in-depth interviews and FDGs, narrative data on perceived barriers to epilepsy care and coping with stigma were utilized to optimize credibility and validity of this qualitative study [14, 15]. We used open–ended questions so as not to limit the range or breadth of discussion among the participants. We positioned the study participants for adequate eye contact with others in the group and the discussion lasted approximately 1 h.

Some of the examples of the open-ended questions used to explore the barriers to managing epilepsy and stigma reduction include; “*What sort of things get in the way of helping you in managing your epilepsy?”* The guide also included examples of follow-up probes *such as “would you explain further”, “please describe what you mean”, and “would you give me an example”.* The interview was recorded, and then transcribed verbatim. Information collected from focus group sessions included interview and observation.

A team of three investigators (MK, HN and MNK) read the focus group transcripts in their entirety to gain familiarity with the data. Segments of text from the transcript were labeled and assigned codes that described meaning of content [13].

The codes were subsequently collapsed into broad themes or categories. MK, HN and MNK independently coded each transcript to ensure consistency and transparency of the coding; discrepancies were resolved by discussion. Finalization of codes was based on the consensus of the qualitative team. We utilized the grounded theory approach to data analysis, involving open, axial and sequential coding, and the constant comparative method to generate constructs (themes) and elaborate the relationship among them [16]. A separate coding dictionary was then constructed for the interviews and focus groups.

## Results

Based on the content of the analyzed study data generated, predominant barriers to epilepsy care were summarized under three themes. Each theme is described and representative quotes illustrating prevailing perceptions are reported in italics. A translator translated all responses in the local language into English.

Three (3) major themes of barriers to epilepsy care emerged: (1) individual level barriers, (2) family and community level barriers, and (3) health care system and provider level barriers.

### Individual level barriers

In Table 1 themes, sub-themes, and key quotations illustrating the perceived individual level barriers to epilepsy care are presented. We grouped these perceived individual level barriers into the 5 subthemes reflecting personal issues, which our respondents identified as barriers in epilepsy care: psychological, behavioral, knowledge, lifestyle and functional impairment.

**Table 1:** Perceived personal level barriers to epilepsy care

Themes and Categories	Illustrative Quotations from Respondents
Psychological barriers	
Psychological distress/PSTD	*“What causes stress is when you get the attack in the presence of your employer and then he tells you, “don’t come to work tomorrow; I don’t want you to die from here.” And remember you need children’s school fees and all the necessities at home. So you end up feeling stressed whether or not you want”.* Respondent PR#1
Behavioral barriers	
Resistance to seeking health care	*“Personally when I had just started my medication, we always argued with my parent at home; I always told her, “I am not an HIV patient to swallow pills daily.” I would get attacks but I didn’t want to swallow medicine. The doctor counseled me, I am now going to two years without a single attack”.* Respondent PR#7:
Medication Non-adherence	*“One might be swallowing the medicine and then someone comes out and advises them to try something else, so they quit the medicine.”* Respondent PR#4
Knowledge barriers	
Causes	*“It seems that thing (illness) is just sent by someone. The way I see this illness, there could be someone who sent it”.* Respondent PR#2:
Cure and prevention	*“That illness isn’t curable. But he has been swallowing medicine for close to 22 years now but he’s still on that medication. These drugs that they give us only enable us to live on”.* Respondent PR#8
Lifestyle barriers	
Alcohol	*“I learnt how to booze from school. So amongst us there could be someone who takes alcohol, but that medicine must not be mixed with alcohol. That is what I learnt”.* Respondent PR#3
Financial constraints	*“My mum was working as a nurse but she was forced to retire, but no one supports us. It is mum who has to find money and yet she doesn’t work anymore. We don’t have any support; we just hustle”.* Respondent PR#2
Functional impairment barriers	*“When he gets an attack, he can spend like three or four days when is just seated at home; he never has any energy or talks.” Respondent CG#24*

#### Mental health barriers

Psychological distress symptoms were frequently reported by PLWE these included; sleep disturbances, mood changes, stress, and post-traumatic stress disorder (PSTD). These participants also reported poor appetite and low energy as well as living in a constant fear of having a seizure.

Respondents reported long episodes of lack of energy after episodes of prolonged attacks:

*When I get an attack, sometimes I can spend a whole week without energy.* Respondent PR#5 (Patient respondent)

For some, just living with the constant fear of having a seizure is so psychologically stressful that it sometimes results in physical symptoms unrelated to the seizure itself:

*“With this illness, suddenly you feel like you are about to have an attack and then get frightened and then the heart beats fast”.* Respondent PR#1

#### Behavioral barriers

The main two behavioral barriers to epilepsy care reported by the respondents were non-adherence to anti-epileptic therapy and difficulties to seeking mainstream medical care. Majority of the respondents reported that they were socially isolated. Some followed the advice of traditional healers, which opposed the use of mainstream medical care.

*“The reason why some people don’t swallow drugs, is that traditional healers say they can treat every illness, so some patients seek treatment there and quit taking their anti-epilepsy medication”.* Respondent PR#19

Both PLWE and caregivers also reported an inability to seek modern medical care and opt for traditional or cultural practices, which impact drug compliance.

*“One could say that ancestral spirits cause the problem and then another person will say that you stepped over charms trapped in the road; so you want to try everything to heal as traditional healers treat the charms and misfortunes”.* Respondent PR#4

Negligence from their caregivers was also reported as one of the leading causes of drug non-adherence.

*“Her mother was negligent about her and would only remember about her from work in the middle of the day and instead return in the evening and then give her both the morning and evening dose at once*”. Respondent PR#11

The study participants also reported reluctance to refill their drugs when feeling better.

*“Well if someone uses medication and realizes that they have improved a little in two months, they think that they have healed so they quit taking it”.* Respondent PR#18

#### Knowledge barriers

For the most part, participants identified the major causes of epilepsy as anger, stress, trauma to the brain, and congenital (inherited/inborn) factors.

While some suggested that becoming angry would damage the brain and cause epilepsy.

*“I think that it could be anger, where someone can get so angry that their vessels swell. It could maybe damage the brain and then get that problem”.* Respondent CG#2:

However, others reported that it is an inborn or inherited illness

*“Some people are born with that illness. For others, I hear that it is a family thing that they just inherit”.* Respondent PR#1

Participants suggested that it is a result of brain injury or comes from the brain.

*“In my childhood when I was still a baby, mum told me that someone was carrying me and I accidentally fell down and spent the next 24 hours unconscious. Then when I was four years old it started”.* Respondent PR#5

However, some of the participants reported epilepsy as being contagious:

*“Sharing things with people who are sick of seizures”.* Respondent CG#3

While others felt it was because of spirits or witchcraft:

“*Ancestral spirits, the first time I got the illness, I saw a ghost and had very long hair and nails, and so I ran. When I looked behind, it waved at me and it was in that night that the illness started”.* Respondent PR#2

However, some PLWE and their caregivers did not know what caused epilepsy:

*“There’s nothing I know about it but what I know is that it kills”.* Respondent PR#17:

While a number of participants felt that epilepsy was preventable and curable:

*“That one is preventable, you can prevent against that illness, the same way you do it for HIV”.* Respondent PR#3:

Others felt differently:

*“That illness doesn’t have a right medicine to treat it. The doctor told us that the medicine that we get to treat that illness only relaxes it but doesn’t cure it. It doesn’t treat the illness to cure it for good”.* Respondent PR#13

#### Lifestyle barriers

Alcohol use and financial constraints were reported as major impediments to epilepsy care and control.

The study participants highlighted alcohol use as a major hindrance to epilepsy care and control,

*“Things got worse because when I took alcohol after swallowing the medicine, they told me to stop”* Respondent PR#2.

As well as being stressed/ashamed when they are talked about or when a seizure occurs in front of family or friends.

*“My biggest challenge is that I do not want to get an attack when my friends are around, I would feel ashamed if am around many people”.* Respondent PR#9

Limitations in self-care as well as participating in home and community activities were also cited as barriers:

*“Sometimes they say; she must not cook. She should never cook for us! What if she falls in the food? Yet I am the one who cooks for the family”* Respondent PR#2.

Participant cited financial constraints to meet the required basic needs and get medications as a major cause of stress in their lives and was reported to be one of the barriers to epilepsy care and management.

*“There is stress about finding money and the children’s school fees also mixes in”.* Respondent PR#10

#### Functional impairment and self-identity barriers

Caregivers highlighted the changes in mood and loss of self -identity in relatives /friends with epilepsy which was attributed to epilepsy.

*“She was easily angered, often started quarrels, and had a short temper. If you are not her mother you cannot stay with her”* Respondent CG#26

In summary, the greatest impediments to epilepsy care cited by the respondents were stress and lack of knowledge regarding epilepsy and epilepsy care. However, other personal barriers included mental health problems, challenges in seeking mainstream medical care, anti-epileptic drug non-adherence, unhealthy lifestyles and functional impairments.

### Family and community level barriers

Table 2 shows the family and community level barriers to epilepsy care with sub-themes and illustrative quotes. Family issues, caregiver burden and lack of community support were the three important subthemes cited by the respondents.

**Table 2:** Perceived family and community level barriers to epilepsy care

Themes and categories	Illustrative quotations from Respondents
Family issues	
Lack of support	*“She came to my home and at that time her grandparent detested her saying, “I won’t be able to manage that child” so she sent her to us and said, “there she is; you should suffer with her but personally I can’t”.”* Respondent CG#22
Caregiver burden	
Lack of information	*“They never explain to you whether it is this or that, the doctor just writes down the drugs”. “They started giving me medicine, so he started on that medication”.* Respondent CG#27
Increased responsibilities	*“Well I have to take care of him such that he doesn’t get an attack in my absence and yet I have a job to attend to. You see it is that job that sustains me so it gets really hard for me. The challenge is with my job. If I am to leave in the morning, I would have to first give him his medicine*”. Respondent CG#24
PTSD	*“When he seizes, I don’t do some things because of stress. I can be like, “no, I don’t even feel energetic”.*” Respondent PR#4
Lack of community support	
Community isolation and attitudes	*“The challenges that I go through especially, people don’t like patients with epilepsy; they fear them. Because whenever he falls down people get scared. Well people especially the family, all have refuted him and instead want me to stay with him. They never treat him well; everyone refutes him and chase him around”.* Respondent CG#24
Lack of epilepsy education programs	*“The problem is that there’s only one care provider; when we come to pick drugs he’s the same person who handles other patients, so maybe he doesn’t get enough time. That is the reason why I said that we need to form a union and provide education to the people and then they choose within themselves peer educators”. Respondent CG#22*

#### Family issues

Lack of family support and stigmatizing behavior by immediate family members were perceived as important barriers to epilepsy care:

*“You have epilepsy so don’t touch my stuff”.* Respondent PR#4:

A barrier to taking epilepsy medications was lack of support or reminders from the family members.

*“You could be living with someone who can’t even remind you to swallow your medicine especially when you are still young”.* Respondent PR#5

Several study participants reported insults or name calling from their family members.

*“At school, I can get just one attack and then my colleagues isolate me. My friend who used to help me, has stopped sharing a seat with me, they say that when they sit with me I would infect them with epilepsy”.* Respondent PR#11

#### Community issues

Stigmatization from the community, as well as from family, compounded the lack of support and increased isolation felt by PLWE:

*“When you have this illness people isolate you a lot and people start saying, “There is a way they call it that sounds so bad, “Epilepsy” or ‘epileptic’, I would prefer a word like convulsions/seizures”.”* Respondent PR#1

#### Lack of community awareness

The study participants felt strongly that there was a lack of attention to educating the community about epilepsy.

*“People used to tell me, “that child is suffering from witchcraft all they advised was trying out several traditional healers,” it seems those people (referring to us) sacrificed that child to the gods’ that we used some dark magic which backfired or that we sacrificed him and that that was why the child was getting those attacks”.* Respondent CG#27

#### Caregiver burden

The study participants were quite vocal about the onerous burden faced in the day-to-day efforts in caring for a PLWE. As shown in Table 2, the lack of correct information about epilepsy care left most of caregivers to resort to ‘trial and error’ methods. This was aggravated by changes in their roles, decreased household income, and increased caregiver responsibilities, often resulting in physical and emotional stress for the caregivers:

The burden of providing all basic needs in life and study participants cited performing most of the chores for the patients.

*“ I have to take care of her because most people find them (patients) disgusting because when it attacks, they drip saliva. Some people think that it is contagious. Of course I don’t feel good because my colleague (partner) who could have supported me, left, am now the only one supporting her”.* Respondent CG#26

### Healthcare system and provider level barriers

Table 3 shows subthemes and quotes describing healthcare system and provider level barriers to epilepsy care. Two important categories of barriers involving access to health care issues and provider issues were identified.

**Table 3:** Perceived healthcare system and provider level barriers to epilepsy care

Financial constraints	“*We don’t have any drugs, so they tell you to go and buy the drugs.”* Respondent PR#5
Location issues	*“The problem with transportation is that we come from far away. Sometimes we would love to come but lack of transport stops us”.* Respondent PR#2
*Health care provider issues*	
Poor communication skills	*“You can meet a care provider who is just too proud! She yelled back at me instead. Maybe she had been angered moments before, but if you are a care provider you don’t respond rudely to patients”.* Respondent PR#3 “*Why should I go to a government hospital to queue up and tell me to buy the medicine”?* Respondent PR#13
Lack of drugs	
Appointment difficulties	*“When we went to hospital, we were told that the doctor who handles such conditions (epilepsy) comes on specific days in a week and yet you have to first make a booking. So hospital looked like a loose end, so we never went back”. Respondent CG#23*
Inadequate disease investigation	*“What I am saying is what the doctor said; they need to first check me up and see what is on the brain”.* Respondent PR#20

### Access to healthcare system issues

Limited health care provider interaction and distance to health centers were major problems, see Table 3. Health facilities were reported to be located far from patients’ homes and majority of people faced challenges in reaching these centers, as either they did not own or had difficulties with out of pocket to meet the transport costs. This was compounded by long waiting times to see a doctor and sometimes being triaged to another hospital:

*“I rarely skip a dose but there are times when I run out of pills and yet I don’t have any money to come here. So you spend days before getting money to come here and then skip some doses.*” Respondent PR# 10

#### Health care provider issues

Table 3 also displays barriers to receiving epilepsy care from health care providers. In addition to appointment difficulties, poor attitudes towards patients, inadequate diagnostic evaluation, and lack of follow up as well as poor written and verbal communications were reported.

Participants reported limited patient-health worker interaction at the health centers they attend.

*“But they don’t give you much time. I used to have … dizziness, whenever I shared it with the doctor, he would respond, “Just swallow your medicine.” When I returned the following day with the same problem he said, “It seems you are not taking your medication” and yet I was swallowing it. But now I am seeing other medicines that I don’t even understand”.* Respondent PR#2

The study participants reported strongly about the need for increasing epilepsy awareness in the community as part of care and stigma reduction efforts. As shown in Table 4, various ideas regarding how to disseminate epilepsy information like holding community education meetings and seminars, door to door visits by health workers, engaging local leaders to spearhead projects, as well as using print and local media to spread the word about epilepsy.

*“I only request the government to get us medicine that is for free”.* Respondent #PR3:

*“To some of us, this illness hinders us from working to raise money. The government should help us and offer the medicine for free since this illness hinders one from working to get money. How will you work if you have that illness?”* Respondent #PR5

Participants suggested the creation of more facilities treating epilepsy to reduce burden.

*“If a fund is available, personally I think it doesn’t matter if they don’t give it to us the people but they create more health facilities in different areas that attend to people with this kind of illness”.* Respondent PR#4

Some proposed refresher courses in disease management for health care providers.

*“The care providers that treat us could be trained maybe some of them are just giving out tablets but don’t know much about the illness”.* Respondent #PR19

Participants reported that they should at least be screened before giving them AEDs and change drugs given where necessary.

*“They should change the medicine and also start testing us to know what kind of illness is in your body”.* Respondent #PR5:

Participants recommended that health care providers treat patients’ politely/well.

*“The care providers should work on their manners too; if I speak to you calmly I don’t want you to yell back. Care providers should work on that”.* Respondent PR#3

Providing counseling and education to caregivers and family members was suggested.

*“I think they should make a meeting with the people that we live with and give them some counseling at first. For some patients that have said that they get stressed, sometimes it is because someone (a caregiver) who yells at them or do other things. So they should find a way of teaching them such that they learn how to handle that patient”.* Respondent PR#14

With a sense of being ignored by the community, study participants recommended a self-help group approach amongst themselves as a way to lessen this sense of isolation felt by PLWE.

**Table 4:** Participants’ recommendations for increasing epilepsy awareness in the community

Themes and categories	Illustrative quotations from respondents
Community meetings/seminars	*“Sensitization is lacking because it could have been on TVs or radios but I have never heard it there. People are ignorant about it (epilepsy). Most people think that one is born with that illness or must been in one’s family lineage not knowing that one can also get it when they are already old. So we need a seminar”.* Respondent PR#3
Provision of anti-epileptic drugs at health centers	*“That illness is not easy because we are similar to HIV patients; it is swallowed for disease control. So I don’t see why they don’t offer us medicine that we can pick free of charge”.* Respondent #PR17
Training care givers	*“There is a need to train the caregivers and I think there should be a group that unites caregivers. So if we the caregivers have a union that fights for their rights and also explain to others that this illness isn’t contagious such that people buy the idea and the community welcomes it, then the patients wouldn’t get isolated anymore. You see in all ways it is only you her caregiver who won’t isolate her but other people have to isolate her if she has epilepsy”.* Respondent CG#27

## Discussion

In community settings, PLWE may be hard to reach, socially isolated, stigmatized and excluded from clinical studies. This qualitative analysis identified and described barriers to epilepsy care in PLWE and their caregivers in a Ugandan population. Findings build upon emerging research and are intended to advance epilepsy care and target underserved individuals in community settings.

The study noted that individual; family/community and health care issues were important barriers to epilepsy care in this study. This is coherent with McLeroy’s social ecological model of health behavior [17], which suggests that behavior, actions, and events, are influenced by individual, interpersonal, organizational, community and policy factors. An understanding of these factors can help with developing appropriate interventions, targeting and addressing these challenges in PLWE.

### Individual factors

The individual factors such as poverty, lack of adequate knowledge, attitudes, stigmatization and coping skills are important issues that impact on epilepsy care and stigma reductions in PLWE and their caregivers. The majority of PLWE in this sample did not know the cause of epilepsy and the associated precipitating factors. Our finding is consistent with earlier studies on epilepsy knowledge and attitudes in Uganda and other similar settings in Africa [4, 18–20]. Having the right knowledge, attitudes and resources to provide adequate epilepsy management in PLWE and their caregivers’ influences the preferred choice of care either traditional healers or mainstream medicine.

PLWE or their caregivers especially in resource limited settings lack the required information about their disease condition including the potential triggers and precipitants of seizures, role of treatment, role of supportive care and the associated anti-epileptic drugs adverse effects [21, 22]. This subsequently may lead to the development of negative attitudes and reduced expectations regarding epilepsy, [19, 23, 24]. Lack of knowledge eventually influences decision-making with respect to AEDs adherence, provision of funds for out of pocket purchase of anti-epileptic drugs by the caretakers and subsequently control of seizures.

### Interpersonal and social support networks

The close social support systems involving the immediate family, occupation group, and friend networks greatly impact on health care seeking behaviors of PLWE and they are in a key position to provide support or guide choices to people living with epilepsy. Health care seeking and self-management behaviors often take place in a shared family environment and in the context of a myriad of relationships that naturally involve individuals beyond the patient [25]. This close interpersonal network provides both general support, such as emotional support, and epilepsy-specific support, including reminders for taking medications, going to doctor appointments and provision of out of pocket for anti-epileptic medications [26]. Lack of this interpersonal support may enhance behaviors like non-medical adherence, failure to seek mainstream medical care and reluctance on refilling their medications especially when they felt better as cited by the study participants. This may be due to inadequate information regarding their disease or resorting to traditional healers within the immediate family members and social support networks [6, 27]. Advocating for increased awareness within caregivers and communities would help address this lack of knowledge. Studies have shown that social support is associated with better self-management and health outcomes particularly when it is encouraging, enhances patient autonomy, or fosters family cohesion [28, 29]. Tailored and focused educational sessions for epilepsy that also includes stress management practices, recognizing symptoms of stress and PTSD, should be incorporated into patient care provision and community outreach programs.

### Institutional factors

In this study, both PLWE and caregivers reported difficulties in accessing health care challenges with the health care providers. Access to health care was mainly related to the distance and transport issues. Majority of the health centers are within towns or cities leaving most of the rural areas within Uganda, underserved. There is a need to create and strengthen the available public-private partnerships within the study area that provide health care and mobilize the districts and country networks that can augment the sharing of knowledge, expansion of existing resources and the ability to generate cost- effective models for inter-sector and cross-site collaborations on epilepsy management. Offering adequate and well-positioned centers within rural areas would attempt to address this. The development of schedules and policies that are adhered to may address the issues of patient contact time and education regarding their illnesses [22]. Refresher trainings of health care providers are urgently needed to replenish the medical knowledge regarding epilepsy management and how to provide psychosocial support with an aim of improving seizure control. Studies have shown that having a positive attitude among health care professionals helps to promote appropriate behavior by patients [30–32].

### Community

Development of a well coordinated and managed systems that establish linkages across sectors of education; health and other health care service providers are urgently needed. This would establish a continuum of services that would nurture health and wellbeing, guaranteeing an approach that support PLWE with adequate epilepsy care. Our findings revealed that stigma was an important obstacle to epilepsy care and is supported by other studies in sub-Saharan Africa [10, 33]. Despite the need of both family and community support for PLWE, the study participants reported being stigmatized, ostracized by their immediate family members. This high level of stigma and lack of support increases stress and impacts on seizure control. The poor knowledge of epilepsy, its causes, prevention and care among the family and community members may be attributed to the stigma [34]. Several study participants reported that epilepsy is contagious through saliva and sharing personal items. More education and community interventions are urgently, needed to address this. The involvement of caregivers of PLWE in education and self-management care interventions can provide appropriate information for epilepsy care.

### Public policy

Epilepsy is generally not recognized as a public health priority, despite the existence of relatively cheap and readily available treatment options [35, 36]. The Abuja declaration adopted by heads of state of African union countries in April 2001 was designed to improve social and economic conditions in the world’s poorest countries by 2015. One of the goals set a target of allocating at least 15% of their annual budget to improve the health sector (http://www.who.int/healthsystems/publications/abuja_declaration/en/). Resources allocations for evidence-based treatments for chronic health conditions that are of relatively modest financial outlay (such as wide-spread use of antiepileptic drugs for epilepsy) may help reduce more costly and crisis-based care (such as accidents and injury associated with poorly controlled seizures). Given the extensive unmet need and barriers to care identified in this study, appropriate and timely access to care for PLWE could positively affect public health and lead to more efficient and impactful use of scarce healthcare resources.

The failure to have sufficient human and financial resources, coupled with unrealistic policy targets continue to impact on the provision of proper and adequate epilepsy management and stigma reduction. Developing policies that allocate resources and implementing them to establish and maintain a coalition that serves a mediating structure connecting individuals living with epilepsy and the larger social environment to create a healthy campus would go a long way in addressing the barriers of epilepsy management.

Finally, addressing the main barrier to epilepsy care, which is, limited epilepsy knowledge in PLWE and their caregivers seems a feasible starting point to improve epilepsy care. Utilizing public health models that have successfully tackled HIV burden and HIV stigma in Uganda would provide an avenue to reduce epilepsy barriers and improve care.

### Limitations

Our study findings on perceived barriers to epilepsy care in PLWE in Uganda have implications for informing policy and care. Notwithstanding, there were some limitations that need to be taken into account. Patients with epilepsy and their caregivers, who receive or seek care in other settings in Uganda, may have different experiences with, and different types of encounters with providers or healthcare systems from our study. The small convenience sample utilized in this study and the conduct of the study in a single urban area in Uganda may limit transferability of the study findings. However, these limitations are offset, to some extent, by the utilization of rigorous qualitative methods described in the study and our use of the Consolidated Criteria for Reporting Qualitative research (COREQ) [37], to improve the rigor, comprehensiveness and credibility of the interviews and focus groups.

## Conclusion

Epilepsy still remains a big problem with multiple barriers to care on a patient, community and health system level. This study in a Ugandan urban shows that epilepsy care efforts targeting the patient, community, and healthcare system levels are needed to address lack of awareness, inadequate or inaccurate knowledge, stigma, poor access, mental health comorbidity and adherence with evidence-based treatments for seizures.

## Data Availability

The datasets used and/or analyzed during the current study are available from the corresponding author on reasonable request.

## Abbreviations

AEDs: Anti-epileptic drugs
FGDs: Focus group discussions
IQR: Inter-quartile range
PLWE: People living with epilepsy
PTSD: Posttraumatic stress disorder
